# Log-normal diameter distribution of Pd-based metallic glass droplet and wire

**DOI:** 10.1038/srep10711

**Published:** 2015-06-01

**Authors:** S. Yaginuma, C. Nakajima, N. Kaneko, Y. Yokoyama, K. S. Nakayama

**Affiliations:** 1 WPI-Advanced Institute for Materials Research, Tohoku University, Sendai 980-8577, Japan; 2 Dexerials Corporation, Tagajyo, Sakuragi 985-0842, Japan; 3 Institute for Materials Research, Tohoku University, Sendai 980-8577, Japan

## Abstract

We have studied the formation of Pd_42.5_Cu_30_Ni_7.5_P_20_ metallic glass droplets and wires in the gas atomization process. We demonstrate that the sizes of droplets and wires can be distinguished by the Ohnesorge number (*O*_*h*_), which is the proportion of the spinnability to the capillary instability, and the diameter distributions follow a log-normal distribution function, implying cascade fragmentation. For droplets, the number significantly increases at *O*_*h*_ < 1 but the diameter gradually decreases. For wires, the number greatly increases at *O*_*h*_ > 1 while the diameter steadies below 400 nm. Further, the wire diameter is quadrupled at *O*_*h*_ = 16 due to the high viscosity which suppresses both capillary breakup and ligament elongation.

Nanostructures of metallic glasses (MGs) have attracted growing interests in materials science and technology. MG nanowires are promising building blocks for magnetic sensors, battery electrodes, and heterogeneous catalysts^1^,^2^,^3^,^4^,^5^,^6^,^7^. The development for practical applications requires a large amount of the high quality MG wires with low cost. Recently, Nakayama *et al.* have succeeded in producing MG nanowires using the gas atomization process that has been a powerful method for producing metal and alloy powders. However, the fragmentation of molten alloys during the gas atomization processes is complicated because it occurs under the non-equilibrium states before solidification. Therefore, the fundamental understanding of fragmentation processes using fluid mechanics is important for controlling sizes and shapes. There have been extensive studies for gas atomization in powder metallurgy^8^. In general, the primary breakup in the atomization creates droplets at the molten alloy surface of melted streams. The secondary breakup produces subsequent droplets and further cascade fragmentation creates fine droplets, which leads to the broad size distribution^10^. However, much less attention has been paid to filamentary structures such as Pele’s (the Hawaiian goddess of volcanoes) hair^11^ though the difference is simply achieved by the spinnability, which is defined as an ability to form into threads or fibers^12^.

The breakup of viscous ligaments through the capillary instability within the time scale *t*_cap_ can be described by^13^


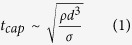


where *d* is the ligament diameter, *σ* is the surface tension, and *ρ* is the density. In contrast, the time scale of the spinnability, *t*_vis_ attributed by the viscous forces is given by


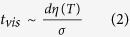


where *η* is the viscosity. Consequently, the Ohnesorge number (*O*_*h*_) is obtained by the ratio of these time scales as^14^
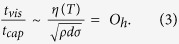


Generally, when *t*_vis_/*t*_cap_ = *O*_*h*_ < 1, the capillary instability dominates and the fragmentation into droplets becomes a favored process. In contrast, when *O*_*h*_ > 1, the spinnability dominates and the ligaments can be stretched before their solidification.

In this paper, we study the diameter distribution of droplets and wires composed of Pd_42.5_Cu_30_Ni_7.5_P_20_ metallic glass (Pd-MG). Pd-MG was used because it has a high glass forming ability and a high resistance to the oxidation^15^. Based on the Angell plot^16^, the viscosity of MGs have been described in the supercooled liquid region, which is defined by the difference between the crystallization temperature (*T*_*x*_) and the grass transition temperature (*T*_*g*_), and the temperature dependence in thermoplastic formability has been discussed, previously^17^,^18^. We focus on the viscosity near the melting point (*T*_*m*_) where it significantly increases when the sample is supercooled from *T*_*m*_. Therefore, the viscosity-dominated process associated with high Ohnesorge number plays an important role in the wire elongation. The aim of this study is fundamental understanding of the formation rule for the MG nanowires and microwires and would be tailoring length, diameter, and distribution. The temperature is a key parameter because it associates with the viscosity. We have created droplets and wires with varying temperature and obtained the diameter distribution by counting them. We found that the distribution follows a log-normal distribution, indicating cascade fragmentation events in the gas atomization. The formation of thicker wires is dominated at *O*_*h*_ > 1 which can be realized only below *T*_*m*_.

## Results

**Figure 1:**
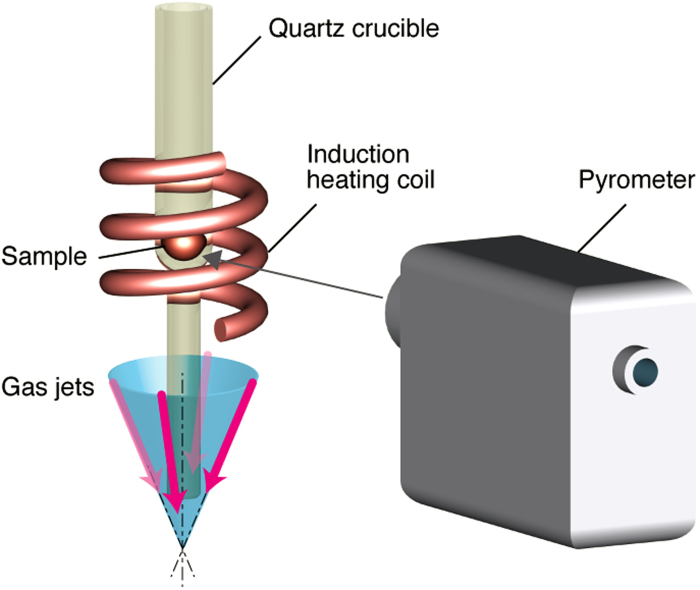
Schematic illustration of the gas atomization. The temperature of a sample was monitored by a pyrometer through the induction heating coil. The angle of gas jets was 45° and the jet speed was adjusted by the pressure. In this study, the Arpressure of jets was 10 MPa.

**Figure 2:**
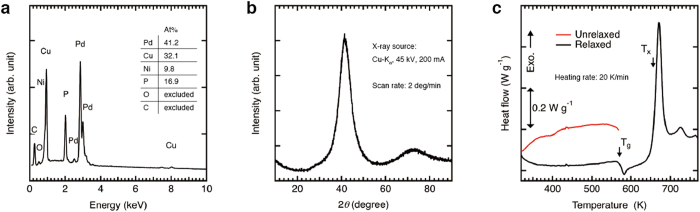
The results of EDX, XRD, and DSC. (a) EDX spectrum of Pd-MG wires created at 773 K. The components of Pd, Cu, Ni, P are equivalent to the mother alloy. (**b**) XRD pattern of Pd-MG products showing broad halo patterns. (**c**) The DSC result shows the significant relaxation in the products. The red curve was measured for as-atomized products up to 570 K below *T*_*g*_. Then, the scans were repeated four times and the black full DSC curve was obtained. The difference between the red and black curves indicates the area of exothermic heat flows.

**Figure 3:**
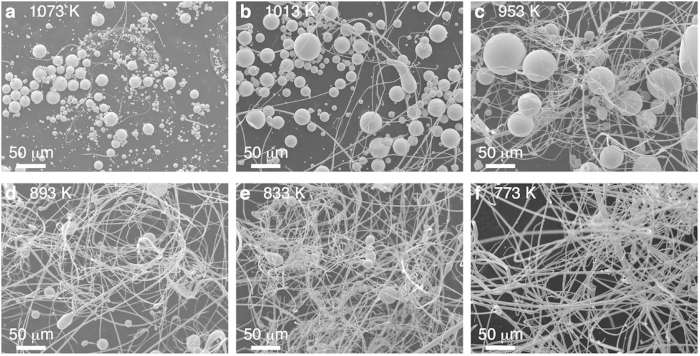
The SEM images of Pd-MG atomized products at (a) *T*_in_ = 1073 K, (b) *T*_in_ = 1013 K, (c) *T*_in_ = 953 K, (d) *T*_in_ = 893 K, (e)*T*_in_ = 833 K, and (f) *T*_in_ = 773 K. All images were taken by the same magnification. The electron beam energy was 15 keV. The products were supported on a carbon tape for imaging.

The Pd-MG sample was heated by an induction heating coil in the gas atomization system (Fig. 1). The induction heating temperature (*T*_in_) is varied as a parameter in this study (See Methods). Figure 2 shows the results of energy dispersive X-ray spectroscopy (EDX), X-ray diffraction (XRD), and differential scanning calorimeter (DSC) for the wires created at *T*_in_ = 773 K. The EDX spectrum in Fig. 2a identify Pd, Cu, Ni, P, C, and O elements, which are equivalent to the original composition, revealing that the incorporation of contaminations during the atomization process is negligible. The gas atomization produces a large amount of droplets and wires that allows conventional XRD and DSC measurements. The XRD pattern is obtained from about 10 mg sample containing mostly wires (cf. Fig. 3f), showing two broad halos corresponding to a fully amorphous phase, as shown in Fig. 2b. The DSC result obtained from about 3 mg of the sample indicates *T*_g_ = 570 K and *T*_x_ = 655 K, as shown in Fig. 2c. These results are consistent with those of bulk Pd-MG^19^ and the glassy phase of the atomized products is sustained. However, there is significant structural relaxation. The red curve in Fig. 2c corresponds to the first scan up to 570 K < *T*_g_. After the first scan, we repeat the scans with four times below 570 K to induce structural relaxation. Then, the DSC curve is obtained up to 770 K, as shown in the black curve in Fig. 2c. The difference between the red curve and the black curve corresponds to the area of exothermic heat flows, indicating that the products significantly include excessive free volume^5^,^6^.

**Figure 4:**
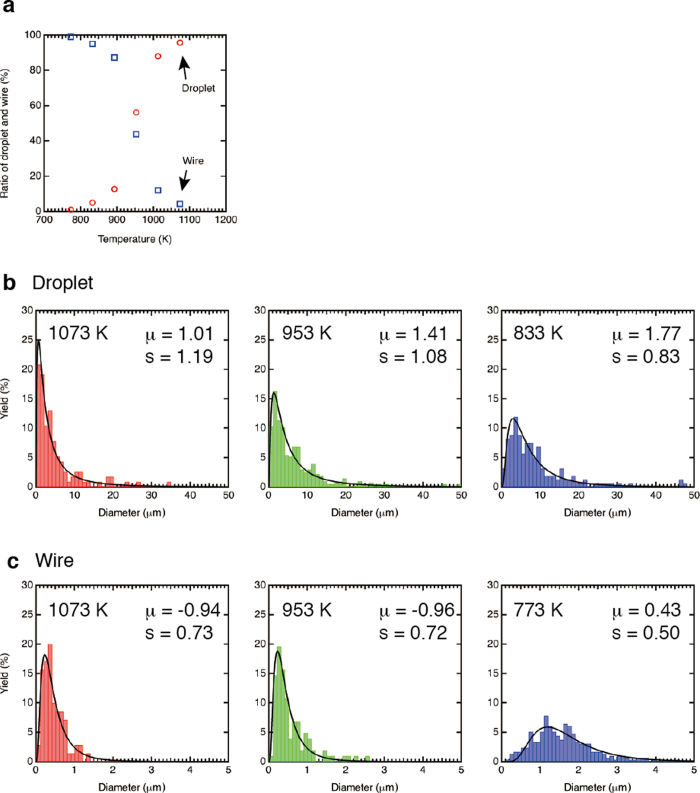
Statistical analyses of SEM images. (a) Ratio of droplets and wires created by different temperatures. There is a crossover at 953 K. (**b**) The diameter distribution of droplets obtained by the different temperature. The diameter increases with decreasing temperature. The solid line is the log-normal distribution function *f*(*d*) to fit the droplet data. The mean (*μ*) and the standard deviation (*s*) are extracted by the fitting curve. (**c**) The diameter distribution of wires. The wire diameter suddenly increases somewhere between 773 K and 833 K (See Table 1).

Figure 3 shows the scanning electron microscopy (SEM) images of droplets and wires that are produced by *T*_*in*_ with the range of 773 ~ 1073 K. Figure 3a shows the image of the product at 1073 K. Droplets are main products, and few wires can be recognized. Figure 3b shows the result obtained at 1013 K, indicating that the number of droplets decreases but the droplet diameter increases. Figure 3c shows the result at 953 K, which produces almost equal numbers of droplet and wire (cf. Fig. 4a). As further decreasing temperature down to *T*_in_ = 773 K in Fig. 3f, few droplets can be seen and the number of wires significantly increases.

To carry out quantitative analyses, we have taken about 20 SEM images for each experimental condition, counted the numbers of 350 ~ 400 for each droplet and wire, and performed statistical analyses. The correlation between droplet and wire is obtained by counting the number of products as a function of the temperature, as shown in Fig. 4a. There is a crossover around *T*_in_ = 953 K. The droplet formation is expected because the superheating above *T*_*m*_ induces the low viscosity that leads to spheroidization. However, the droplet diameter obtained at 953 K increases as compared with the result obtained at 1073 K. Such tendency also appears to the wires where the diameter increases with decreasing temperature. Figure 4b and 4c show the diameter distribution of droplets and wires, respectively. The vertical axis is the yield that is obtained by the number of corresponding product (droplet or wire) diameters divided by the total number of each product. The lateral axis is the diameters that have a range of 50 nm ~ 7.0 μm. It shows that the median diameter of wires is about one order smaller than that of droplet. We employ the log-normal distribution function to fit the droplet and wire data and extract the mean (*μ*) and the standard deviation (*s*) in the diameter distribution. The log-normal distribution is accepted as one of the important functions for fragmentation processes in atomization^9^,^20^. When a probability distribution forms a log-normal distribution as a function of *d*, it follows a normal distribution as a function of ln *d*. Generally, *f*(*d*) is defined as:


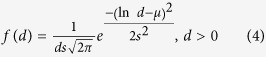


**Figure 5:**
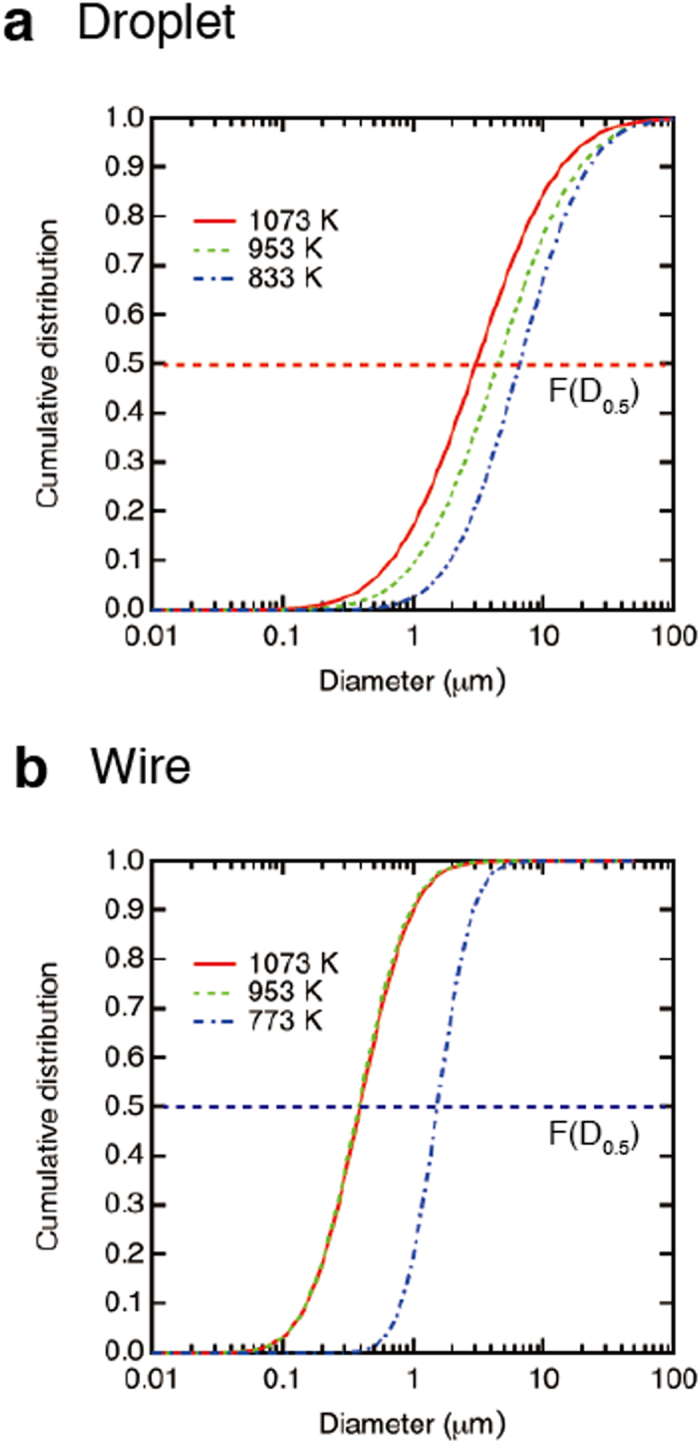
The cumulative number distribution *F*(*d*) curves (a) of droplets at 833, 953, and 1073 K and (b) of wires at 773, 953, and 1073 K. The dashed line corresponds to *F*(*D*_0.5_) = 0.5 and the median diameter *D*_0.5_ can be obtained at different temperatures.

The solid curves in Fig. 4b and 4c represent the fitted log-normal distribution function and the extracted values of *μ* and *s* are labeled. For the size distribution in the statistical analysis, we employ the median diameter *D*_0.5_ in the cumulative distribution function *F*(*d*) because it can describe a representative value in the asymmetric distribution. *F*(*d*) is described as^9^


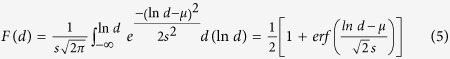


where *erf* (*x*) is the error function that is written by 
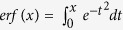
. Figure 5 represents the cumulative distribution curves of droplets at 833, 953, and 1073 K and of wires at 773, 953, and 1073 K using the values of *μ* and *s* obtained in Fig. 4. *D*_0.5_ is defined as the line of *F*(*D*_0.5_) = 0.5, which is shown as the dashed line in Fig. 5, and consequently, *D*_0.5_ = *e*^*μ*^. Table 1 shows the summarized results of *D*_0.5_ obtained at each temperature. For the droplet, *D*_0.5_ = 6.52 μm at 833 K gradually decreases to *D*_0.5_ = 3.03 μm at 1073 K. For the wire, *D*_0.5_ = 1.53 μm at 773 K suddenly decreases to *D*_0.5_ = 0.40 μm at 833 K and the values of *D*_0.5_ are almost saturated to *D*_0.5_ = 0.39 μm at 1073 K. The thicker wires at 773 K imply that the high viscosity suppresses not only capillary breakup but also ligament elongation.

## Discussion

The results obtained by the statistical analyses are important because they can provide median diameter as evaluation criteria. To understand our experimental data, we have calculated the Ohnesorge number that depends on the temperature as a function of *d*. For the calculations, the Vogel-Fulcher-Tammann (VFT) relation is applied to the viscosity, *η*(*T*) = *η*_0_ exp{D*T_0_/(*T* - *T*_0_)}, where *η*_0_ is the viscosity at infinite temperature, D^*^ is a measurement of the kinetic fragility, and *T*_0_ is the VFT temperature^16^. From equation (3),


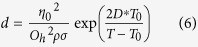


**Figure 6:**
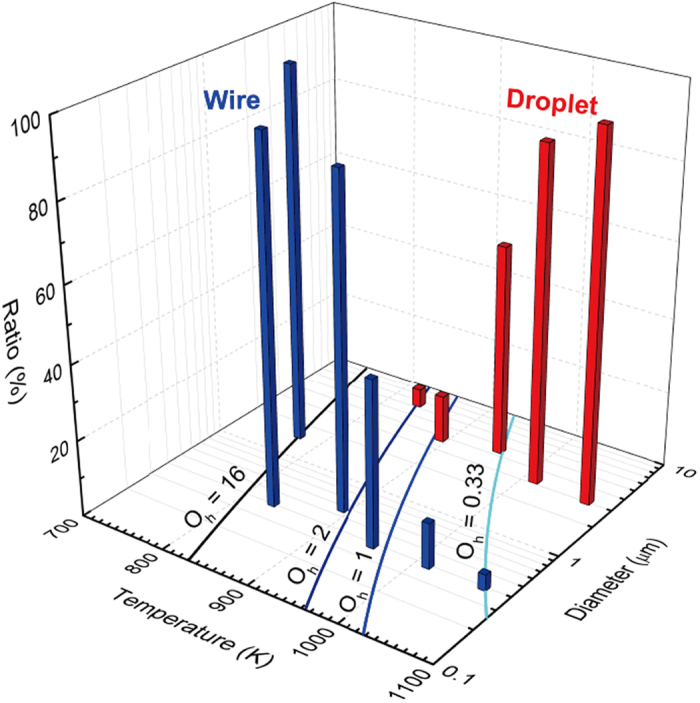
The Ohnesorge curves at *O*_h_ = 0.33, 1, 2, and 16 are obtained by the VFT conditions, where the diameter increases with decreasing temperature. The temperature-dependent median diameters in Table 1 are combined with the droplet and wire ratios as the vertical axis. The ratio of droplets significantly decreases at *O*_h_ < 1. In contrast, the ratio of wires greatly increases at *O*_h_ > 1.

Assuming *σ* = 1 N/m, we use the values of *η*_0_ = 4 × 10^−5^ Pa s, D* = 12, and *T*_0_ = 365 K from refs. 17 and 18. We also take account of the temperature dependent *ρ* in the Pd-MG as *ρ* = {1.304 × 10^−8^ *T* + 1.10 × 10^−4^}^−1^ from ref. 21. Figure 6 shows the Ohnesorge curves at *O*_h_ = 0.33, 1, 2, and 16, indicating the tendency where the diameter increases with decreasing temperature. The median diameters in Table 1 and the droplet and wire ratios in Fig. 4a are superimposed in Fig. 6. The ratio of droplets significantly decreases at *O*_h_ < 1 and it is only 5% (833 K) at *O*_h_ = 2. In contrast, the ratio of wires greatly increases at *O*_h_ > 1 where it increases from 12% at 1013 K to 44% at 953 K. Furthermore, the sudden increase of diameter to 1.53 μm at 773 K corresponds to *O*_*h*_ = 16. In this stage, the high viscosity suppresses ligament elongation and thus thicker wires dominantly appear.

The experimental results demonstrate that the diameters obey the log-normal distribution that brings us perspectives for theoretical understanding of the atomization process. The log-normal distribution of diameters is originally derived from Kolmogorov’s concept of viewing breakup as a discrete random process^22^,^23^. According to the concept, the median is obtained from the products that were produced at the final stage in breakup-cascade processes. To explain the experimental results, it would be necessary to provide a novel phenomenological model beyond the Kolmogorov’s concept. In our experimental situation, the viscosity effect is extremely strong, as seen at *O*_h_ > 2. Furthermore, the droplets are rapidly solidified during the deformation process. In contrast, the wire deformation is influenced by both viscosity and solidification. Smoluchowski has also expressed the break-up processes of viscoelastic liquid with a gamma distribution^24^,^25^. The difference between the gamma distribution and the log-normal distribution can be identified by the tail in distribution shape. In details, the gamma distribution is derived by taking into account the coalescence of small droplets. However, the coalescences should be a rare event in our experiment because they are rapidly solidified. Thus, the argument on the basis of the *D*_0.5_ fitted by the log-normal distribution would be plausible.

To account for the ratio of droplets and wires theoretically, we require further analysis based not only on the diameter distributions but also on wire lengths. Some non-trivial phenomena are still expected to mediate both fragmentation and elongation. For example, the onset of elongation at a droplet occurs when the externally applied force, *f  *^(ex)^, which should be from the gas jets, exceeds the capillary force, *f  *^(cap)^. The external force depends on the surface area of a melting droplet. Namely, it is proportional to the square of the characteristic length of a droplet, *f  *^(ex)^ = *α*_1_*pd*^2^, where *p* is the external pressure which is assumed as a given constant and *α*_1_ is just a numerical constant. While, the capillary force against the elongation is proportional to the characteristic length itself, *f  *^(cap)^ = *α*_2_*σd*, where *α*_2_ is the numerical constant. Hence, there is a critical diameter *d*_c_ beyond which a droplet can be elongated. The critical diameter is proportional to the capillary coefficient and inversely proportional to the external pressure, *d*_c_ = (*α*_2_/*α*_1_)*σ*/*p*. In addition, a droplet is also subjected to the viscous force while elongated. If there is some timescale while which the droplet is subjected to the external force, the final length of the wire (including the droplet) is determined by the viscous force.

In summary, we have studied the formation rule of Pd-MG droplets and wires. The size evaluation of gas atomized products has succeeded with the median diameters which are derived by fitting of the log-normal distribution function and the half value in the cumulative distribution function. We show that the Ohnesorge curves describe the temperature dependence on the diameter which increases with decreasing temperature and the appearance of droplets and wires can be expressed by the Ohnesorge number. There is an implication from this study. To obtain fine nanowires, the condition should be *O*_h_ > 2, but the much higher Ohnesorge number leads to a thicker wire. Further, the jet speed that is related to the gas jet pressure during atomization must be investigated^26^.

## Methods

### Materials Fabrication

We used a custom-built gas atomization system constructed by Nisshin Giken Co. and the experiments were carried out for 5 gram at maximum for alloys or metals. In this experiment, about 3 g of Pd-MG alloy in a quartz crucible was heated by an induction heating system in Ar atmosphere. The end of the quartz crucible had a nozzle with the diameter of 0.1 mm. *T*_in_ was monitored by an optical pyrometer (CHINO, IR-CAI3CS) with the emissivity of 0.50, which was calibrated by the melting point of Al in the gas atomization system, as illustrated in Fig. 1. The sample was once heated up to *T*_in_ = 1073 K well above the melting point (*T*_m_ = 803 K)^15^. The melt stream was extruded through the nozzle with the injection Ar pressure of 0.05 MPa, and was atomized with the Ar gas jet pressure of 10 MPa. After the atomization, all products were accumulated in the bottom of the chamber and the cyclone collector. The total lost of the products was within 5 weight %.

### Materials characterization

Structural and compositional analyses were performed with scanning electron microscopy (JEOL JSM-7800F), energy dispersive X-ray spectroscopy (Oxford Instruments X-Max 50), and X-ray diffraction (Rigaku SmartLab 9MTP). The thermal properties were measured by differential scanning calorimeter (Rigaku DSC8230), which was carried out with the heating rate of 20 K/min. For the statistical analysis, we used OriginPro 9.1 (OriginLab). For the analysis of droplet and wire diameters, imGauge (Imsoft Co., Ltd.) was used.

## Additional Information

**How to cite this article**: Yaginuma, S. *et al.* Log-normal diameter distribution of Pd-based metallic glass droplet and wire. *Sci. Rep.* **5**, 10711; doi: 10.1038/srep10711 (2015).

## Figures and Tables

**Table 1:** Median diameter D_0.5_ (μm) of droplets and wires at different temperature.

Temperature (K)	773	833	893	953	1013	1073
Droplet	-	6.52	3.81	4.47	3.20	3.03
Wire	1.53	0.40	0.56	0.38	0.39	0.39
